# Breast density reduction as a predictor for prognosis in premenopausal women with estrogen receptor-positive breast cancer: an exploratory analysis of the updated ASTRRA study

**DOI:** 10.1097/JS9.0000000000000907

**Published:** 2023-11-22

**Authors:** Soong June Bae, Hee Jeong Kim, Hyun-Ah Kim, Jai Min Ryu, Seho Park, Eun-Gyeong Lee, Seock-Ah Im, Yongsik Jung, Min Ho Park, Kyong Hwa Park, Su Hwan Kang, Eunhwa Park, Sung Yong Kim, Min Hyuk Lee, Lee Su Kim, Anbok Lee, Woo Chul Noh, Sungchan Gwark, Seonok Kim, Joon Jeong

**Affiliations:** a Department of Surgery, Gangnam Severance Hospital; b Institute for Breast Cancer Precision Medicine, Yonsei University College of Medicine; c Division of Breast, Department of Surgery, Asan Medical Center, University of Ulsan College of Medicine; d Department of Surgery, Korea Cancer Center Hospital, Korea Institute of Radiological and Medical Sciences; e Division of Breast Surgery, Department of Surgery, Samsung Medical Center, Sungkyunkwan University School of Medicine; f Division of Breast Surgery, Department of Surgery, Yonsei Cancer Center, Yonsei University College of Medicine; g Department of Clinical Epidemiology and Biostatistics, Asan Medical Center; h Seoul National University Hospital, Cancer Research Institute, Seoul National University, College of Medicine; i Department of Surgery, Ewha Womans University College of Medicine, Ewha Womans University Mokdong Hospital; j Department of Surgery, Konkuk Universitiy Medical Center; k Korea University Anam Hospital, Department of internal medicine, Division of Medical Oncology/Hematology; l Department of Surgery, Soonchunhyang University Hospital, Seoul; m Department of Surgery, Dong-A University Hospital, Dong-A University College of Medicine, Busan; n Department of Surgery, Soonchunhyang University Cheonan Hospital, Cheonan; o Yeungnam University College of Medicine, Daegu; p Department of Surgery, Chung-Ang University Gwangmyeong Hospital, Gwangmyeong; q Department of Surgery, Chonnam National University Medical School and Chonnam National University Hwasun Hospital, Gwangju; r Department of Surgery, Ajou University, School of Medicine, Suwon; s Center for Breast Cancer, Research Institute and Hospital, National Cancer Center, Goyang, South Korea

**Keywords:** breast neoplasm, density, endocrine therapy, estrogen receptor-positive, ovarian function suppression

## Abstract

**Background:**

While the relationship between mammographic breast density reduction (MDR) and endocrine therapy efficacy has been reported in estrogen receptor (ER)-positive breast cancer, it is still unclear in premenopausal women, especially in the case of adding ovarian function suppression (OFS) to antihormone therapy. The authors investigated the impact of MDR on prognosis stratified by treatment based on the updated results of the ASTRRA trial.

**Materials and methods:**

The ASTRRA trial, a randomized phase III study, showed that adding OFS to tamoxifen (TAM) improved survival in premenopausal women with estrogen receptor-positive breast cancer after chemotherapy. The authors updated survival outcomes and assessed mammography before treatment and the annual follow-up mammography for up to 5 years after treatment initiation. Mammographic density (MD) was classified into four categories based on the Breast Imaging-Reporting and Data System. MDR-positivity was defined as a downgrade in MD grade on follow-up mammography up to 2 years after randomization, with pretreatment MD grade as a reference.

**Results:**

The authors evaluated MDR in 944 of the 1282 patients from the trial, and 813 (86.2%) had grade III or IV MD. There was no difference in the MDR-positivity rate between the two treatment groups [TAM-only group (106/476 (22.3%)) vs. TAM+OFS group (89/468 (19.0%)); *P*=0.217). MDR-positivity was significantly associated with better disease-free survival (DFS) in the TAM+OFS group (estimated 8-year DFS: 93.1% in MDR-positive vs. 82.0% in MDR-negative patients; HR: 0.37; 95% CI: 0.16–0.85; *P*=0.019), but not in the TAM-only group (*P*_interaction_=0.039). MDR-positive patients who received TAM+OFS had a favorable DFS compared to MDR-negative patients who received only TAM (HR: 0.30; 95% CI: 0.13–0.70; *P*=0.005).

**Conclusion:**

Although the proportion of MDR-positive patients was comparable between both treatment groups, MDR-positivity was independently associated with favorable outcomes only in the TAM+OFS group.

## Introduction

Highlights

- In young premenopausal patients, mammographic breast density reduction was identified in ~20%, regardless of adjuvant endocrine therapy.
- Mammographic breast density reduction was significantly associated with favorable long-term survival outcomes in the tamoxifen plus ovarian function suppression group, but not in the tamoxifen alone group.
- The monitoring changes in mammographic breast density have potential clinical utility to predict the effectiveness of adjuvant endocrine therapy plus ovarian function suppression in premenopausal women.

Adjuvant endocrine therapy is one of the most effective systemic adjuvant treatment options for premenopausal women with estrogen receptor (ER)-positive breast cancer^1,2^. The Suppression of Ovarian Function Trial (SOFT), Tamoxifen and Exemestane Trial (TEXT) adjuvant trials, and Adding Ovarian Suppression to Tamoxifen for Premenopausal Breast Cancer (ASTRRA) trial showed that the addition of ovarian function suppression (OFS) to adjuvant endocrine therapy improved survival outcomes in premenopausal patients with ER-positive breast cancer^3,4^. Nevertheless, premenopausal women are usually considered at a higher risk than postmenopausal women^5^, and some premenopausal patients with ER-positive breast cancer who have high-risk factors experience recurrence over a long follow-up period^6^. Moreover, the incidence of premenopausal breast cancer is rising worldwide, particularly in high-income countries^7^. Accordingly, identifying the predictors of endocrine therapy response that can be easily applied in clinical practice would greatly benefit this group.

Mammographic breast density (MD), which reflects the extent of fibroglandular tissue in the breast, is a known predictor of breast cancer risk^8,9^. Amassing evidence has shown consistent results, indicating that breast cancer risk is high in cases of high MD among the general population^10,11^. In high-risk women receiving tamoxifen (TAM) for primary prevention, MD decline was significantly related to a reduced breast cancer risk^12–14^. Endocrine therapy, such as TAM or aromatase inhibitors (AIs), induces mammographic breast density reduction (MDR)^15^; it is expected that this parameter can be applied to predict endocrine therapy efficacy. Given that breast density is usually higher in premenopausal women than in postmenopausal women^16,17^, the clinical usefulness of MDR seems more pronounced in premenopausal women. Although the predictive role of MDR in endocrine therapy in premenopausal breast cancer has been retrospectively evaluated for decades, inconsistent results have been identified across the studies^18–21^. Furthermore, the value of monitoring MDR after initiating adjuvant endocrine treatment has not been evaluated in phase III clinical trials. In addition, evidence is insufficient on whether adding OFS to TAM is more likely to induce MDR and whether MDR can predict the benefit of adding OFS to TAM.

The ASTRRA trial compared 5-year TAM plus 2-year OFS with 5-year TAM-only in premenopausal women in the Republic of Korea, and the primary results were reported previously^4^. In this exploratory analysis of the ASTRRA trial, we aimed to clarify the relationship between MDR and clinical outcomes in premenopausal women with ER-positive breast cancer to explore whether MDR is a predictor of treatment efficacy in each treatment group (TAM-only vs. TAM+OFS).

## Methods

### Study population

ASTRRA trial (ClinicalTrials.gov identifier: NCT00912548) was an investigator-initiated, open-label, prospective, randomized, multicenter, phase III study evaluating the efficacy of adding OFS to TAM in women aged 45 years or younger with ER-positive breast cancer who remained in a premenopausal state or resumed ovarian function after chemotherapy. The eligibility criteria and methodology have been previously described^4^. The patients were enrolled within 3 months of the last chemotherapy, and TAM was prescribed for all patients at the time of enrollment. Ovarian function status was determined based on the serum follicle-stimulating hormone levels or evidence of vaginal bleeding. When patients were premenopausal at enrollment or evaluated as having resumed ovarian function within 2 years after enrollment, they were randomly assigned to complete 5 years of TAM alone [group B (resumed ovarian function) or D (premenopausal status at enrollment)] or 5 years of TAM with OFS every 4 weeks for 2 years [group C (resumed ovarian function) or E (premenopausal status at enrollment)]. Patients who continued to have chemotherapy-induced amenorrhea for 2 years from the time of enrollment were categorized into the permanent menopause group (group A) and were not included in the primary analysis. In this study, we separately analyzed the impact of MDR on prognosis in group A. The prespecified primary endpoint was 5-year disease-free survival (DFS), and the secondary endpoint was 5-year overall survival (OS) in the intention-to-treat (ITT) population.

This study reports on a follow-up investigation of the ASTRRA trial, which included 35 institutions. Data were obtained from 33 of these institutions, while data from the first 5 years of the original trial were used for the remaining two institutions that did not participate in the follow-up study. Data collection was carried out between July and December 2021, covering the period from enrollment in the ASTRRA trial to the last follow-up or death. Follow-up data were gathered through medical chart reviews. The study was conducted in accordance with the strengthening the reporting of cohort, cross-sectional and case–control studies in surgery (STROCSS) criteria^22^ (Supplemental Digital Content 1, http://links.lww.com/JS9/B375) and registered on Research Registry (resarchregistry9552). This study was approved by the institutional review boards of all participating institutions, and a waiver for informed consent was granted.

The exploratory analysis reported herein was performed in a Breast Density Cohort with available mammograms to evaluate the predictive role of MDR. We retrospectively collected MD grade information measured on preoperative mammography and mammography performed at 1-year intervals for up to 5 years after surgery. We updated the survival outcomes with a median follow-up period of 109 months.

### Mammographic breast density

We collected data for breast density grade from digitalized mammographic images performed annually for up to 5 years after the initial breast cancer diagnosis. The breast density grade was measured on the unaffected contralateral breast using digitalized mammographic images by radiologists at each institution. Mammographic density (MD) patterns were classified into four grades according to Breast Imaging-Reporting and Data System (BI-RADS) categories: (1) grade I, almost entirely fat (<25% glandular); (2) grade II, scattered fibroglandular densities (25–50% glandular); (3) grade III, heterogeneously dense (50–75% glandular); and (4) grade IV, extremely dense (>75% glandular). Since the participants were randomly assigned to TAM alone for 5 years or TAM for 5 years with OFS for 2 years in the ASTRRA trial, we evaluated the MDR with the baseline mammogram taken before surgery and the follow-up mammograms taken up to 2 years after randomization. MDR-positivity was defined as a decrease in breast density grade at least once during the follow-up mammograms, with the baseline MD as a reference.

### Outcomes

The major objective of this exploratory analysis was to assess prognosis according to MDR stratified by treatment group. DFS was defined as the time from enrollment to the first event of invasive local recurrence, regional recurrence, distant recurrence, invasive contralateral breast cancer, secondary malignancy, or death for any reason. OS was defined as the time from enrollment to the first death event for any reason. Recurrence-free survival (RFS) was defined as the time from enrollment to the first event of invasive local recurrence, regional recurrence, distant recurrence, or death for any reason. Distant metastasis-free survival (DMFS) was defined as the time from enrollment to the first event of distant recurrence or death for any reason. Locoregional recurrence-free survival (LRRFS) was defined as the time from enrollment to the first event of invasive local recurrence, regional recurrence, or death for any reason.

We also compared survival outcomes as defined from the time of randomization to the time of events to exclude the impact of MDR due to chemotherapy-induced amenorrhea on prognosis. In this case, the MD measured on mammography performed immediately before random assignment rather than before surgery was defined as the baseline MD. A decline in the breast density grade, at least once during the follow-up mammograms up to 2 years after randomization, was classified as MDR-positivity.

### Statistical analysis

Discrete variables between the groups were compared using the χ^2^test or Fisher’s exact test. The Kaplan–Meier method was used to estimate the survival rate, and the results between the groups were compared using the log-rank test. The hazard ratio (HR) with its associated 95% CI was estimated using the Cox regression model adjusted for key baseline prognostic factors (age, tumor size, lymph node status, tumor grade, and HER2 status). Interaction terms for each survival outcome between MDR (positive vs. negative) and treatment regimens (TAM-only vs. TAM+OFS) were considered. Statistical significance tests were two-sided, and a *P* value <.05 was considered statistically significant.

## Results

### Patient characteristics

**Figure 1:**
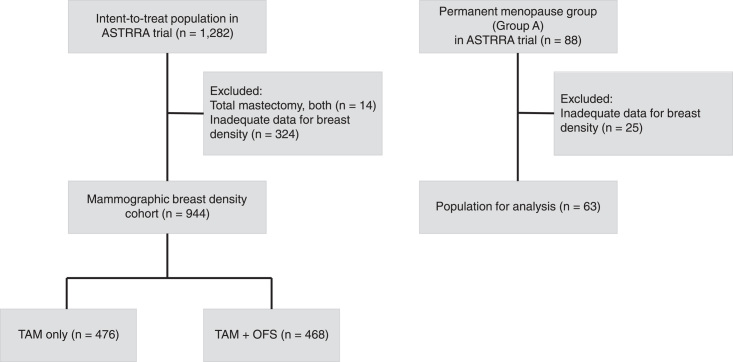
Study diagram.

MDR was successfully evaluated in the Breast Density Cohort of 944 patients, which is 73.6% of the 1282 patients in the ASTTRA ITT population. Of the 944 patients, 476 belonged to the TAM-only group, and 468 belonged to the TAM+OFS group (Fig. 1). The baseline characteristics of the Breast Density Cohort were comparable to those of the ASTTRA ITT population (Table 1). Overall, 555 (58.8%) patients were 40–45 years old; 496 (52.5%) patients had lymph node metastasis, 147 (15.6%) patients had HER2-positive breast cancer, and 486 (51.5%) patients had tumor grade 2. Most patients (86.2%) had MD grades III or IV at baseline.

**Table 1:** Patient characteristics.

	No. (%)			
Characteristics	ASTTRA cohort (*n*=1282)	Breast density cohort (*n*=944)	*P*
Age at enrollment, years			
<35	172 (13.4)	131 (13.9)	0.788
35–39	367 (28.6)	258 (27.3)	
40–45	743 (58.0)	555 (58.8)	
Lymph node status			0.252
Negative	577 (45.0)	448 (47.5)	
Positive	705 (55.0)	496 (52.5)	
Tumor size, cm			0.499
<2	617 (48.1)	468 (49.6)	
≥2	665 (51.9)	476 (50.4)	
Tumor grade			0.890
1	206 (16.1)	146 (15.5)	
2	663 (51.7)	486 (51.5)	
3	305 (23.8)	224 (23.7)	
Unknown	108 (8.4)	88 (9.3)	
HER2 status			0.423
Negative	776 (60.5)	551 (58.4)	
Positive	176 (13.7)	147 (15.6)	
Unknown	330 (25.7)	246 (26.1)	
Chemotherapy regimen			0.889
Anthracycline plus cyclophosphamide	378 (29.5)	302 (32.0)	
Anthracycline plus cyclophosphamide followed by taxane	652 (50.9)	471 (49.9)	
Anthracycline plus taxane	58 (4.5)	42 (4.4)	
Anthracycline plus cyclophosphamide and taxane	13 (1.0)	9 (1.0)	
Fluorouracil, anthracycline, and cyclophosphamide	148 (11.5)	95 (10.1)	
Other taxane-based regimens	13 (1.0)	11 (1.2)	
Other nontaxane-based regimens	9 (0.7)	8 (0.8)	
Unknown	11 (0.9)	6 (0.6)	
Surgery			0.330
Total mastectomy	504 (39.3)	350 (37.1)	
Breast-conserving surgery	743 (58.0)	560 (59.3)	
Unknown	35 (2.7)	34 (3.6)	
Radiotherapy at time of enrollment			0.401
Done	720 (56.2)	547 (57.9)	
Not done	562 (43.8)	397 (42.1)	
Baseline mammographic breast density grade			NA
I	—	17 (1.8)	
II	—	114 (12.1)	
III	—	534 (56.6)	
IV	—	279 (29.6)	

HER2, human epidermal growth factor receptor 2; NA, not available.

Of 944 patients in the Breast Density cohort, 195 (20.7%) were MDR-positive. There was no difference in the prevalence rate of MDR-positivity between the TAM-only group (22.3%) and the TAM+OFS group (19.0%, *P*=0.217, Supplement Fig. 1A, Supplemental Digital Content 2, http://links.lww.com/JS9/B376). The MDR-positivity rate increased with higher baseline MD grade: grade IV (32.3%), grade III (16.9%), and grade II (13.2%). The MDR-positivity rate was not significantly different across treatment groups stratified by the baseline MD grade (Supplement Table 1, Supplemental Digital Content 2, http://links.lww.com/JS9/B376).

The characteristics of the patients in the treatment group according to MDR stratification are described in Table 2. Compared with the MDR-negative group, there was a higher proportion of grade 1 tumors in the MDR-positive group (11.6 vs. 19.8% in the TAM-only group, *P*=0.039, and 14.2 vs. 31.5% in the TAM+OFS group, *P*=0.002). However, no evidence of an association between MDR and other characteristics was found in the TAM-only and TAM+OFS groups.

**Table 2:** Patient characteristics according to MDR in breast density cohort.

	TAM-Only (*n*=476)			TAM + OFS (*n*=468)		
	No. (%)				No. (%)		
Characteristics	MDR-negative (*n*=370)	MDR-positive (*n*=106)	*P*	MDR-negative (*n*=379)	MDR-positive (*n*=89)	*P*
Age at enrollment, years						
<35	49 (13.2)	11 (10.4)	0.732	61 (16.1)	10 (11.2)	0.507
35–39	103 (27.8)	31 (29.2)		100 (26.4)	24 (27.0)	
40–45	218 (58.9)	64 (60.4)		218 (57.5)	55 (61.8)	
Lymph node status			0.979			0.203
Negative	174 (47.0)	50 (47.2)		176 (46.4)	48 (53.9)	
Positive	196 (53.0)	56 (52.8)		203 (53.6)	41 (46.1)	
Tumor size, cm			0.063			0.933
<2	195 (52.7)	45 (42.5)		185 (48.8)	43 (48.3)	
≥2	175 (47.3)	61 (57.5)		194 (51.2)	46 (51.7)	
Tumor grade			0.039			0.002
1	43 (11.6)	21 (19.8)		54 (14.2)	28 (31.5)	
2	197 (53.2)	61 (57.5)		190 (50.1)	38 (42.7)	
3	96 (25.9)	18 (17.0)		94 (24.8)	16 (18.0)	
Unknown	34 (9.2)	6 (5.7)		41 (10.8)	7 (7.9)	
HER2 status			0.760			0.423
Negative	212 (57.3)	65 (61.3)		223 (58.8)	51 (57.3)	
Positive	58 (15.7)	15 (14.2)		57 (15.0)	17 (19.1)	
Unknown	100 (27.0)	26 (24.5)		99 (26.1)	21 (23.6)	
Chemotherapy regimen			0.737^a^			0.292^a^
Anthracycline plus cyclophosphamide	118 (31.9)	30 (28.3)		128 (33.8)	26 ( (29.2)	
Anthracycline plus cyclophosphamide followed by taxane	187 (50.5)	52 (49.1)		189 (49.9)	43 (48.3)	
Anthracycline plus taxane	14 (3.8)	6 (5.7)		19 (5.0)	3 (3.4)	
Anthracycline plus cyclophosphamide and taxane	6 (1.6)	1 (0.9)		1 (0.3)	1 (1.1)	
Fluorouracil, anthracycline, and cyclophosphamide	34 (9.2)	15 (14.2)		33 (8.7)	13 (14.6)	
Other taxane-based regimens	4 (1.1)	1 (0.9)		5 (1.3)	1 (1.1)	
Other nontaxane-based regimens	4 (1.1)	0 (0.0)		2 (0.5)	2 (2.3)	
Unknown	3 (0.8)	1 (0.9)		2 (0.5)	0 (0.0)	
Surgery			0.266			0.482
Total mastectomy	130 (35.1)	45 (42.5)		145 (38.3)	30 (33.7)	
Breast-conserving surgery	225 (60.8)	59 (55.7)		219 (57.8)	57 (64.0)	
Unknown	15 (4.1)	2 (1.9)		15 (4.0)	2 (2.2)	
Radiotherapy at time of enrollment			0.049			0.420
Done	228 (61.6)	54 (50.9)		218 (57.5)	47 (52.8)	
Not done	142 (38.4)	52 (49.1)		161 (42.5)	42 (47.2)	

a *P*-values are obtained with Fisher’s exact test.

HER2, human epidermal growth factor receptor 2; MDR, mammographic breast density reduction; TAM + OFS, tamoxifen plus ovarian function suppression group; TAM-only, tamoxifen-only group.

### Prognosis according to MDR

**Figure 2:**
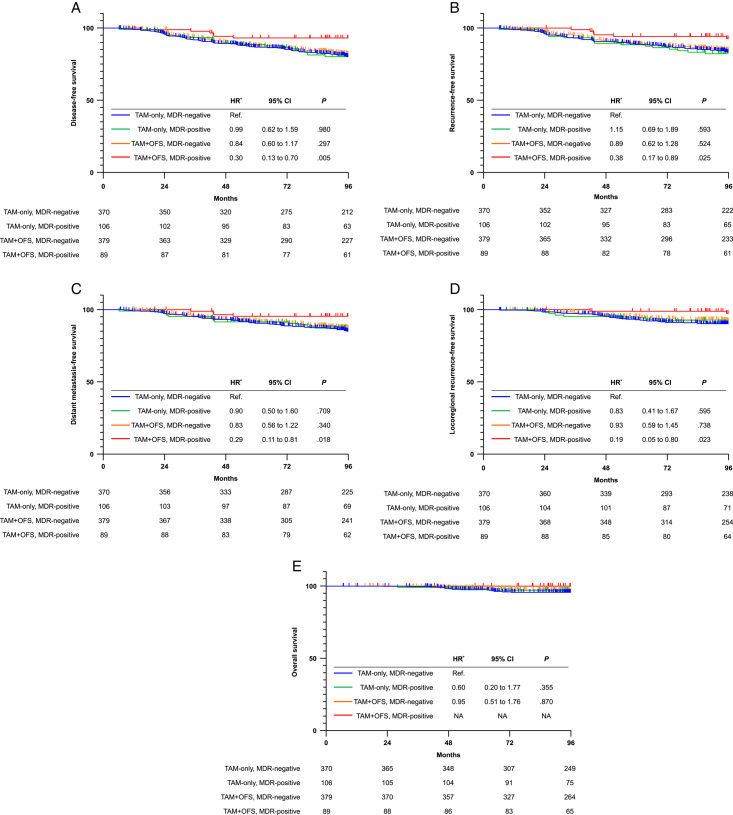
Kaplan–Meier estimates of (A) disease-free survival, (B) recurrence-free survival, (C) distant metastasis-free survival, (D) locoregional recurrence-free survival, and (E) overall survival according to breast density reduction (MDR) and treatment groups. HR, hazard ratio; TAM-only, tamoxifen-only group; TAM + OFS, tamoxifen plus ovarian function suppression group. *Hazard ratio with its associated 95% CI was estimated using the Cox regression model adjusted for age, tumor size, lymph node status, tumor grade, and HER2 status.

During the median follow-up of 109 months, the prognosis according to MDR was different in each treatment group. There was no difference in the survival rate according to MDR in the TAM-only group, whereas MDR-positive patients in the TAM+OFS group had a favorable prognosis (Table 3). In the TAM+OFS group, the 8-year DFS rate was 82.0% in MDR-negative patients and 93.1% in MDR-positive patients (HR, 0.37; 95% CI: 0.16–0.85; *P*=0.019). A significant interaction between MDR and the treatment group for DFS (*P*_*interaction*_=0.039) was detected. We confirmed similar results for RFS (HRa, 0.43; 95% CI: 0.18–1.00, *P*=0.060), DMFS (HR, 0.35; 95% CI: 0.12–0.97, *P*=0.043), and LRRFS (HR, 0.21; 95% CI: 0.05–0.86, *P*=0.030). In addition, there were no deaths among MDR-positive patients in the TAM+OFS group. When performing combined analysis with MDR and treatment group, significantly favorable survival outcomes were identified only in MDR-positive patients who received TAM+OFS compared to MDR-negative patients who received TAM-only (Fig. 2): DFS (HR, 0.30; 95% CI: 0.13–0.70, *P*=0.005), RFS (HR, 0.38; 95% CI: 0.17–0.89, *P*=0.025), DMFS (HR, 0.29; 95% CI: 0.11–0.81, *P*=0.018), and LRRFS (HR, 0.19; 95% CI: 0.05–0.80, *P*=0.023).

**Table 3:** Hazard ratio and estimates of survival according to mammographic breast density reduction stratified by treatment groups.

	TAM-only (*n*=476)				TAM + OFS (*n*=468)				
Survival	MDR	8-year survival rate	HR^a^ (95% CI)	*P*	MDR	8-year survival rate	HR^a^ (95% CI)	*P*	*P*_*interaction*_
DFS	MDR-negative (*n*=370)	80.2%	Ref.		MDR-negative (*n*=379)	82.0%	Ref.		0.039
	MDR-positive (*n*=106)	80.2%	1.03 (0.64–1.63)	0.917	MDR-positive (*n*=89)	93.1%	0.37 (0.16–0.85)	0.019	
OS	MDR-negative (*n*=370)	95.6%	Ref.		MDR-negative (*n*=379)	96.4%	Ref.		NA
	MDR-positive (*n*=106)	97.1%	0.88 (0.33–2.39)	0.809	MDR-positive (*n*=89)	100%	NA	NA	
RFS	MDR-negative (*n*=370)	83.7%	Ref.		MDR-negative (*n*=379)	85.0%	Ref.		0.054
	MDR-positive (*n*=106)	82.3%	1.19 (0.73–1.95)	0.489	MDR-positive (*n*=89)	92.7%	0.43 (0.18–1.00)	0.050	
DMFS	MDR-negative (*n*=370)	85.0%	Ref.		MDR-negative (*n*=379)	87.5%	Ref.		0.108
	MDR-positive (*n*=106)	87.2%	0.97 (0.55–1.72)	0.924	MDR-positive (*n*=89)	95.4%	0.35 (0.12–0.97)	0.043	
LRRFS	MDR-negative (*n*=370)	90.2%	Ref.		MDR-negative (*n*=383)	92.3%	Ref.		0.081
	MDR-positive (*n*=106)	91.1%	0.96 (0.49–1.89)	0.900	MDR-positive (*n*=87)	97.4%	0.21 (0.05–0.86)	0.030	

a Hazard ratio with its associated 95% CI was estimated using the Cox regression model adjusted for age, tumor size, lymph node status, tumor grade, and HER2 status.

DFS, disease-free survival; DMFS, distant metastasis-free survival; HR, hazard ratio; LRRFS, locoregional-free survival; MDR, mammographic breast density reduction; OS, overall survival; RFS, recurrence-free survival; TAM + OFS, tamoxifen plus ovarian function suppression group; TAM-only, tamoxifen-only group.

We investigated the relationship between MDR and survival outcomes in 551 patients with a confirmed HER2-negative status. The proportion of MDR-positive patients was 23.5% in the TAM-only group and 18.6% in the TAM+OFS group (*P*=0.162, Supplement Fig. 1B, Supplemental Digital Content 2, http://links.lww.com/JS9/B376). Similarly, MDR was not significantly associated with prognosis in the TAM-only group. Meanwhile, the 8-year DFS in the TAM+OFS group was 80.6% in MDR-negative patients and 93.9% in MDR-positive patients (HR, 0.30; 95% CI: 0.09–0.97; *P*=0.044). We detected a significant interaction between MDR and treatment groups for DFS (*P*_*interaction*_=0.033). Similar trends were observed for RFS and DMFS, although the differences were not statistically significant. No locoregional recurrence or deaths occurred among MDR-positive patients in the TAM+OFS group (Supplement Table 2, Supplemental Digital Content 2, http://links.lww.com/JS9/B376). In addition, MDR-positive patients who received TAM+OFS had better DFS than MDR-negative patients who received TAM-only (HR, 0.25; 95% CI: 0.08–0.80, *P*=0.019, Supplement Table 3, Supplemental Digital Content 2, http://links.lww.com/JS9/B376). In addition, We analyzed the impact of MDR on prognosis stratified by treatment in 147 patients with HER2-positive status (Supplement Table 4, Supplemental Digital Content 2, http://links.lww.com/JS9/B376) and 246 patients with HER2-unknown status (Supplement Table 5, Supplemental Digital Content 2, http://links.lww.com/JS9/B376), respectively. Overall, we found similar trends in each subpopulation although the association between MDR and prognosis stratified by treatment was not statistically significant.

Next, we compared survival outcomes defined as the period from the random assignment to the time of the first occurrence of MDR in each treatment group. This analysis was performed on 910 patients with a median follow-up of 108 months, excluding those whose MD grade on mammography immediately before random assignment could not be evaluated. The 8-year DFS in the TAM+OFS group was 81.0% in MDR-negative patients and 94.1% in MDR-positive patients (HR, 0.18; 95% CI: 0.11–0.82, *P*=0.046). We also detected a significant interaction between MDR and treatment groups for DFS (*P*_*interaction*_=0.046). Similar results were observed for other survival outcomes. Meanwhile, MDR was not significantly associated with clinical outcomes in the TAM-only group (Supplement Table 6, Supplemental Digital Content 2, http://links.lww.com/JS9/B376).

### Permanent menopause group

Data for MDR and extended survival outcomes were collected from 63/88 (71.6%) patients in the permanent menopause group (group A) from the ASTRRA trial (Fig. 1). The MDR-positivity rate of the 63 patients was 22.2%. Because the data for the characteristics of patients belonging to group A were not collected in the ASTRRA trial, we could not analyze the clinicopathologic factors according to MDR in this subpopulation. During the median follow-up of 107 months, the 8-year DFS, OS, RFS, DMFR, and LRRFS in MDR-negative patients were 88.7, 97.8, 90.9, 93.2, and 92.9%, respectively. There were no events in 14 MDR-positive patients, but these differences were not statistically significant (Supplement Table 7, Supplemental Digital Content 2, http://links.lww.com/JS9/B376).

## Discussion

In this exploratory analysis of the ASTRRA trial with extended follow-up data, we explored the characteristics and clinical outcomes according to MDR depending on the treatment groups. High breast density is common in premenopausal women, especially Asians^23^, and more than 80% of patients had high breast density of grade III or higher in this study. A decline in breast density was observed in ~20% of patients, regardless of the treatment group. Interestingly, the MDR provided independent prognostic information on survival outcome in the TAM+OFS group, whereas the MDR was not associated with prognosis in the TAM-only group. In line with this, we found that MDR-positive patients had a better prognosis in the permanent menopause group (group A).

Our findings suggest the potential to tailor treatment strategies based on MDR. Specifically, MDR-positive patients who received TAM+OFS demonstrated a favorable prognosis, indicating that MDR can serve as a predictive factor for sensitivity to endocrine plus OFS. In contrast, MDR-negative patients displayed a relatively worse prognosis, with an 8-year DFS rate of 82%, despite receiving chemotherapy followed by endocrine therapy in addition to OFS. There may be a need to explore treatment-escalation options, such as incorporating CDK4/6 inhibitors or extending endocrine therapy, for this specific subgroup. Further evidence is required to apply MDR as a component of personalized therapy in a clinical context.

There were three concerns: i) MDR-positive patients had a low tumor grade, ii) the patients with HER2-positive or unclear HER2 status were included in our cohort, and iii) when MD at preoperation was defined as a baseline value, chemotherapy-induced amenorrhea may have affected the MDR regarding the survival rate in groups B and C, which were not randomized immediately after enrollment to allow for recovery of ovarian function. However, MDR remained a strongly significant factor after adjusting for prognosis-related variables, including tumor grade. Furthermore, we confirmed homogenous results when restricted to patients with confirmed HER2-negative status or when survival was defined as the period from randomization to the time of the first event.

The prognostic implications of MDR identified in the TAM+OFS group may be explained by the serum estradiol level, which is associated with mammary ductal hyperplasia and breast cancer development. TAM blocks the effect of circulating estrogen by competing with estradiol at the receptor site^24^, while OFS, such as gonadotropin-releasing hormone agonists, suppress estrogen synthesis in the ovaries, lowering circulating estradiol levels to that of postmenopausal women^25^. TAM+OFS (goserelin or buserelin) effectively suppressed serum estradiol levels, but TAM-only did not^26,27^. Our results suggest that MDR-positivity may be a potential biosensor of OFS for circulating estrogen deprivation regarding clinical outcomes in premenopausal women with ER-positive breast cancer. However, we could not assess whether the change in serum estradiol levels was associated with MDR in the TAM+OFS group. Further studies are required to verify this issue.

Unlike in the TAM+OFS group, MDR was not related to survival benefits in the TAM-only group. Consistent with our findings, several previous studies have shown that MDR does not predict the benefits of TAM without OFS in premenopausal women^18,19,21^. In contrast, one study reported that MDR during adjuvant TAM therapy without OFS was independently associated with a favorable prognosis in premenopausal patients with ER-positive breast cancer^20^. Here, the mean age of the patients was 45.3 years (SD, 7.6); hence, more than half were in their late 40s. Most patients received chemotherapy (71.6%), and chemotherapy administration was significantly associated with MDR. Accordingly, chemotherapy-induced OFS may occur in substantial patients, which may have affected the outcome. Nevertheless, why MDR is meaningless in relation to clinical outcomes in the TAM-only group is questionable. Numerous factors, including age^28^, heredity^29,30^, lifestyle^31^, hormone therapy^32^, and RANK/RANKL^33,34^, have potential associations with breast density; however, there is no consensus on the influence of clinically meaningful MDR. Consequently, explaining our findings requires a better understanding of the biological basis of breast density.

Our study had several limitations. First, our results should be interpreted with caution because this exploratory study was not predefined in the statistical analysis plan. In addition, the ASTRRA trial was conducted in only one country of a single ethnicity. Thus, validation in an independent cohort including other races is required. Second, the patients with HER2-positive or unknown status were included in this analysis, which may affect our findings. Although the results were not statistically significant due to the small number of patients, we also found similar trends in these subpopulations. Moreover, we confirmed the robust results in patients with HER-negative breast cancer. Third, we determined MDR-positivity or MDR-negativity based on visual assessment with the BI-RADs classification, which was routinely recorded during mammography. Although this density grade is a semiqualitative method depending on the radiologists^35,36^, a central review was not performed in the present study. Furthermore, applying a semiqualitative method for assessing MD may have caused a similar MDR-positivity rate between the TAM-only and TAM+OFS groups. New semi-automated and automated density quantitative assessments have been developed^37–39^. Several density assessment tools incorporating deep learning algorithms have also been developed with promising results^40,41^. Applying these new technologies may allow for a more objective and accurate assessment of the likelihood of MDR in predicting the efficacy of adjuvant endocrine therapy.

Finally, in the era of SOFT and TEXT trials, AI plus OFS has emerged as a new option in adjuvant endocrine therapy in premenopausal women, and it is expected to be more widely applied in clinical practice. However, we could not assess whether MDR reflects the efficacy of AI plus OFS because this subpopulation was not included in the ASTRRA trial. Further studies are warranted to determine the relationship between MDR after treatment with AIs plus OFS and breast cancer outcomes. In addition, the major difference between the ASTRRA trial and the SOFT and TEXT trials is the administration period of OFS (2 years in the ASTRRA trial versus 5 years in the SOFT and TEXT trials). Future studies are needed to evaluate whether MDR is a helpful marker for determining the optimal OFS duration.

## Conclusion

In summary, this is the first study to assess the impact of a decline in breast density on prognosis stratified by adjuvant endocrine therapy (TAM-only vs. TAM+OFS) in young women less than 45 years from a well-designed phase III trial. Patients who experienced MDR had substantially better long-term survival in the TAM+OFS group but not in the TAM-only group. The present findings support the importance of assessing breast density changes to evaluate the effectiveness of adjuvant endocrine therapy plus OFS in premenopausal women with ER-positive breast cancer. These results need to be externally validated.

## Ethical approval

The study was conducted in line with the ethical guidelines of the Declaration of Helsinki, and approved by the institutional review boards of all participating institutions.

## Consent

The need for informed consent was waived under the approval of the IRB due to its retrospective design.

## Source of funding

This research was supported by a grant from the Korea Health Technology R&D Project through the Korea Health Industry Development Institute (KHIDI), funded by the Ministry of Health & Welfare, Republic of Korea (grant number: HI19C0481, HC19C0147).

## Author contribution

S.J.B.: conceptualization, data curation, formal analysis, investigation, methodology, writing – original draft, and writing – review and editing; H.J.K.: conceptualization, data curation, formal analysis, funding acquisition, investigation, writing – original draft, and writing – review and editing; H.-A.K., J.M.R., S.P., E.-G.L., Y.J., M.H.P., S.H.K., E.P., A.L., S.G., S.K.: data curation, formal analysis, investigation, and methodology; S.-A.I., K.H.P., S.Y.K., M.H.L., L.S.K., W.C.N.: data curation, formal analysis, investigation, methodology, and supervision; J.J.: conceptualization, formal analysis, investigation, methodology, supervision, writing – original draft, and writing – review and editing. All authors approved the final version of the manuscript.

## Conflicts of interest disclosure

The authors of this work have nothing to disclose.

## Research registration unique identifying number (UIN)

1. Name of the registry: Research Registry.
2. Unique identifying number or registration ID: researchregistry 9552.
3. Hyperlink to your specific registration (must be publicly accessible and will be checked): https://www.researchregistry.com/register-now#home/registrationdetails/65112fcdc719ee0029180d50.

## Guarantor

Soong June Bae, Hee Jeong Kim, and Joon Jeong.

## Data availability statement

The datasets generated and analyzed during the current study are available from the corresponding author on request.

## Presentation

This work was Presented at the general poster session of 2022 American Society of Clinical Oncology Annual Conference, Chicago, IL, June 3-7, 2022.

## Provence and peer review

Not commissioned, externally peer-reviewed.

## Supplementary Material
