# Supplementary material for: Breast density reduction as a predictor for prognosis in premenopausal women with estrogen receptor-positive breast cancer: an exploratory analysis of the updated ASTRRA study
Source: Int J Surg. 2023 Nov 22;110(2):934–42. doi: 10.1097/JS9.0000000000000907 (PMC10871609; doi:10.1097/JS9.0000000000000907)

**Supplementary Table 1. Proportion of MDR-positivity according to baseline mammographic breast density stratified by treatment group.**

| Breast density grade | TAM only | | TAM + OFS | | Total | | *P* value |
| --- | --- | --- | --- | --- | --- | --- | --- |
|  | No. | % | No. | % | No. | % |  |
| II | 8 of 53 | 15.1 | 7 of 61 | 11.5 | 15 of 114 | 13.2 | .569 |
| III | 50 of 275 | 18.2 | 40 of 259 | 15.4 | 90 of 534 | 16.9 | .398 |
| IV | 48 of 138 | 34.8 | 42 of 141 | 29.8 | 90 of 279 | 32.3 | .372 |

Abbreviations: MDR, mammographic breast density reduction; TAM only, tamoxifen-only group; TAM + OFS, tamoxifen plus ovarian function suppression group.

**Supplementary Table 2. Hazard ratio and estimates of survival according to mammographic breast density reduction stratified by treatment groups in patients with confirmed HER2-negative breast cancer.**

| Survival | TAM only (n = 277) | | | | TAM + OFS (n = 274) | | | | *P_interaction_* |
| --- | --- | --- | --- | --- | --- | --- | --- | --- | --- |
|  | MDR | 8-year survival rate | HR^*^ (95% CI) | *P* value | MDR | 8-year survival rate | HR^*^ (95% CI) | *P* value |  |
| DFS | MDR-negative (n = 212) | 81.3% | Ref. |  | MDR-negative (n = 223) | 80.6% | Ref. |  | .033 |
|  | MDR-positive (n = 65) | 78.8% | 1.15 (0.64 to 2.06) | .637 | MDR-positive (n = 51) | 93.9% | 0.30 (0.09 to 0.97) | .044 |  |
| OS | MDR-negative (n = 212) | 95.9% | Ref. |  | MDR-negative (n = 223) | 94.4% | Ref. |  | NA |
|  | MDR-positive (n = 65) | 96.8% | 1.09 (0.34 to 3.51) | .887 | MDR-positive (n = 51) | 100% | NA | NA |  |
| RFS | MDR-negative (n = 212) | 84.3% | Ref. |  | MDR-negative (n = 223) | 83.5% | Ref. |  | .036 |
|  | MDR-positive (n = 65) | 80.6% | 1.36 (0.74 to 2.51) | .323 | MDR-positive (n = 51) | 93.9% | 0.34 (0.10 to 1.10) | .073 |  |
| DMFS | MDR-negative (n = 212) | 85.0% | Ref. |  | MDR-negative (n = 223) | 85.5% | Ref. |  | .100 |
|  | MDR-positive (n = 65) | 83.8% | 1.23 (0.63 to 2.41) | .547 | MDR-positive (n = 51) | 93.9% | 0.39 (0.12 to 1.29) | .123 |  |
| LRRFS | MDR-negative (n = 212) | 91.0% | Ref. |  | MDR-negative (n = 223) | 90.3% | Ref. |  | NA |
|  | MDR-positive (n = 65) | 91.9% | 1.08 (0.45 to 2.56) | .868 | MDR-positive (n = 51) | 100% | NA | NA |  |

^*^Hazard ratio with its associated 95% confidence interval was estimated using the Cox regression model adjusted for age, tumor size, lymph node status, and tumor grade.

Abbreviations: HR, hormone receptor; HER2, human epidermal growth factor receptor 2; Abbreviations: TAM only, tamoxifen-only group; TAM + OFS, tamoxifen plus ovarian function suppression group; MDR, mammographic breast density reduction; HR, hazard ratio; CI, confidential interval; DFS, disease-free survival; OS, overall survival; RFS, recurrence-free survival; DMFS, distant metastasis-free survival; LRRFS, locoregional-free survival.

**Supplementary Table 3. Hazard ratio of survival outcomes and 95% confidential intervals associated with a decrease in breast density compared with no decrease in patients with HR+HER2- breast cancer**

|  |  | Adjusted HR^*^ | | |
| --- | --- | --- | --- | --- |
| Survival | Breast density | HR | 95% CI | *P* value |
| DFS | TAM-only, MDR-negative | 1.00 | Ref |  |
|  | TAM-only, MDR-positive | 1.18 | 0.66 to 2.09 | .582 |
|  | TAM + OFS, MDR-negative | 0.85 | 0.55 to 1.30 | .453 |
|  | TAM + OFS, MDR-positive | 0.25 | 0.08 to 0.80 | .019 |
| OS | TAM-only, MDR-negative | 1.00 | Ref |  |
|  | TAM-only, MDR-positive | 1.08 | 0.34 to 3.43 | .893 |
|  | TAM + OFS, MDR-negative | 1.43 | 0.66 to 3.08 | .366 |
|  | TAM + OFS, MDR-positive | NA | NA |  |
| RFS | TAM-only, MDR-negative | 1.00 | Ref |  |
|  | TAM-only, MDR-positive | 1.39 | 0.76 to 2.56 | .285 |
|  | TAM + OFS, MDR-negative | 0.94 | 0.59 to 1.49 | .785 |
|  | TAM + OFS, MDR-positive | 0.32 | 0.10 to 1.04 | .058 |
| DMFS | TAM-only, MDR-negative | 1.00 | Ref |  |
|  | TAM-only, MDR-positive | 1.21 | 0.62 to 2.37 | .569 |
|  | TAM + OFS, MDR-negative | 0.94 | 0.58 to 1.53 | .797 |
|  | TAM + OFS, MDR-positive | 0.37 | 0.11 to 1.22 | .103 |
| LRRFS | TAM-only, MDR-negative | 1.00 | Ref |  |
|  | TAM-only, MDR-positive | 1.10 | 0.46 to 2.61 | .834 |
|  | TAM + OFS, MDR-negative | 1.21 | 0.68 to 2.18 | .516 |
|  | TAM + OFS, MDR-positive | NA | NA |  |

^*^Hazard ratio with its associated 95% confidence interval was estimated using the Cox regression model adjusted for age, tumor size, lymph node status, and tumor grade.

Abbreviations: TAM + OFS, tamoxifen plus ovarian function suppression group; HR, hazard ratio; CIs, confidential intervals; MDR, mammographic breast density reduction; DFS, disease-free survival; RFS, recurrence-free survival; BCFI, breast cancer-free interval; DMFS, distant metastasis-free survival; LRRFS, locoregional-free survival.

**Supplementary Table 4. Hazard ratio and estimates of survival according to mammographic breast density reduction stratified by treatment groups in patients with HER2-positive status.**

| Survival | TAM only (n = 73) | | | | TAM + OFS (n = 74) | | | | *P_interaction_* |
| --- | --- | --- | --- | --- | --- | --- | --- | --- | --- |
|  | MDR | 8-year survival rate | HR^*^ (95% CI) | *P* value | MDR | 8-year survival rate | HR^*^ (95% CI) | *P* value |  |
| DFS | MDR-negative (n = 58) | 79.8% | Ref. |  | MDR-negative (n = 57) | 78.8% | Ref. |  | .419 |
|  | MDR-positive (n = 15) | 80.0% | 0.28 (0.56 to 7.20) | .282 | MDR-positive (n = 17) | 88.2% | 0.48 (0.15 to 1.60) | .234 |  |
| OS | MDR-negative (n = 58) | 92.9% | Ref. |  | MDR-negative (n = 57) | 96.2% | Ref. |  | NA |
|  | MDR-positive (n = 15) | 93.3% | 0.97 (0.05 to 19.3) | .986 | MDR-positive (n = 17) | 100% | NA | NA |  |
| RFS | MDR-negative (n = 58) | 83.8% | Ref. |  | MDR-negative (n = 57) | 82.6% | Ref. |  | .502 |
|  | MDR-positive (n = 15) | 80.0% | 3.09 (0.75 to 12.83) | .120 | MDR-positive (n = 17) | 85.6% | 1.23 (0.22 to 6.93) | .813 |  |
| DMFS | MDR-negative (n = 58) | 81.8% | Ref. |  | MDR-negative (n = 57) | 88.4% | Ref. |  | .932 |
|  | MDR-positive (n = 15) | 93.3% | 1.35 (0.21 to 8.60) | .748 | MDR-positive (n = 17) | 100.0% | NA | NA |  |
| LRRFS | MDR-negative (n = 58) | 85.8% | Ref. |  | MDR-negative (n = 57) | 88.4% | Ref. |  | .886 |
|  | MDR-positive (n = 15) | 80.0% | 3.03 (0.65 to 14.11) | .157 | MDR-positive (n = 17) | 85.6% | 1.38 (0.23 to 8.38) | .729 |  |

^*^Hazard ratio with its associated 95% confidence interval was estimated using the Cox regression model adjusted for age, tumor size, lymph node status, and tumor grade.

Abbreviations: TAM only, tamoxifen-only group; TAM + OFS, tamoxifen plus ovarian function suppression group; MDR, mammographic breast density reduction; HR, hazard ratio; CI, confidential interval; DFS, disease-free survival; OS, overall survival; RFS, recurrence-free survival; DMFS, distant metastasis-free survival; LRRFS, locoregional-free survival.

**Supplementary Table 5. Hazard ratio and estimates of survival according to mammographic breast density reduction stratified by treatment groups in patients with HER2-unknown status.**

| Survival | TAM only (n = 126) | | | | TAM + OFS (n = 120) | | | | *P_interaction_* |
| --- | --- | --- | --- | --- | --- | --- | --- | --- | --- |
|  | MDR | 8-year survival rate | HR^*^ (95% CI) | *P* value | MDR | 8-year survival rate | HR^*^ (95% CI) | *P* value |  |
| DFS | MDR-negative (n = 100) | 78.4% | Ref. |  | MDR-negative (n = 99) | 86.5% | Ref. |  | .719 |
|  | MDR-positive (n = 26) | 83.6% | 0.72 (0.22 to 2.39) | .282 | MDR-positive (n = 21) | 95.2% | 0.30 (0.04 to 2.28) | .243 |  |
| OS | MDR-negative (n = 100) | 96.7% | Ref. |  | MDR-negative (n = 99) | 97.8% | Ref. |  | NA |
|  | MDR-positive (n = 26) | 100% | NA | NA | MDR-positive (n = 21) | 100% | NA | NA |  |
| RFS | MDR-negative (n = 100) | 82.6% | Ref. |  | MDR-negative (n = 99) | 89.5% | Ref. |  | .863 |
|  | MDR-positive (n = 26) | 88.3% | 0.91 (0.22 to 3.73) | .897 | MDR-positive (n = 21) | 95.2% | 0.39 (0.05 to 3.05) | .371 |  |
| DMFS | MDR-negative (n = 100) | 87.1% | Ref. |  | MDR-negative (n = 99) | 91.5% | Ref. |  | .867 |
|  | MDR-positive (n = 26) | 92.3% | 0.69 (0.12 to 3.81) | .668 | MDR-positive (n = 21) | 95.2% | 0.56 (0.07 to 4.56) | .591 |  |
| LRRFS | MDR-negative (n = 100) | 91.1% | Ref. |  | MDR-negative (n = 99) | 95.8% | Ref. |  | NA |
|  | MDR-positive (n = 26) | 95.8% | 0.57 (0.07 to 4.95) | .606 | MDR-positive (n = 21) | 100% | NA | NA |  |

^*^Hazard ratio with its associated 95% confidence interval was estimated using the Cox regression model adjusted for age, tumor size, lymph node status, and tumor grade.

Abbreviations: TAM only, tamoxifen-only group; TAM + OFS, tamoxifen plus ovarian function suppression group; MDR, mammographic breast density reduction; HR, hazard ratio; CI, confidential interval; DFS, disease-free survival; OS, overall survival; RFS, recurrence-free survival; DMFS, distant metastasis-free survival; LRRFS, locoregional-free survival.

**Supplementary Table 6. Hazard ratio and estimates of survival defined as from the randomization to the time of first event according to mammographic breast density reduction stratified by treatment groups.**

| Survival | TAM only (n = 459) | | | | TAM + OFS (n = 451) | | | | *P_interaction_* |
| --- | --- | --- | --- | --- | --- | --- | --- | --- | --- |
|  | MDR | 8-year survival rate | HR^*^ (95% CI) | *P* value | MDR | 8-year survival rate | HR^*^ (95% CI) | *P* value |  |
| DFS | MDR-negative (n = 378) | 78.2% | Ref. |  | MDR-negative (n = 382) | 81.0% | Ref. |  | .046 |
|  | MDR-positive (n = 81) | 80.5% | 0.99 (0.58 to 1.69) | .970 | MDR-positive (n = 69) | 94.1% | 0.18 (0.11 to 0.82) | .018 |  |
| OS | MDR-negative (n = 378) | 95.9% | Ref. |  | MDR-negative (n = 382) | 94.3% | Ref. |  | NA |
|  | MDR-positive (n = 81) | 96.1% | 0.92 (0.30 to 2.76) | .875 | MDR-positive (n = 69) | 100% | NA | NA |  |
| RFS | MDR-negative (n = 378) | 81.5% | Ref. |  | MDR-negative (n = 382) | 83.8% | Ref. |  | .053 |
|  | MDR-positive (n = 81) | 81.9% | 1.19 (0.68 to 2.08) | .553 | MDR-positive (n = 69) | 93.5% | 0.35 (0.13 to 0.97) | .044 |  |
| DMFS | MDR-negative (n = 378) | 83.8% | Ref. |  | MDR-negative (n = 382) | 86.1% | Ref. |  | .167 |
|  | MDR-positive (n = 81) | 86.9% | 0.91 (0.47 to 1.77) | .784 | MDR-positive (n = 69) | 95.5% | 0.32 (0.10 to 1.05) | .060 |  |
| LRRFS | MDR-negative (n = 378) | 90.2% | Ref. |  | MDR-negative (n = 382) | 89.9% | Ref. |  | .060 |
|  | MDR-positive (n = 81) | 89.8% | 1.08 (0.51 to 2.27) | .842 | MDR-positive (n = 69) | 98.0% | 0.13 (0.18 to 0.97) | .046 |  |

^*^Hazard ratio with its associated 95% confidence interval was estimated using the Cox regression model adjusted for age, tumor size, lymph node status, tumor grade, and HER2 status.

Abbreviations: TAM only, tamoxifen-only group; TAM + OFS, tamoxifen plus ovarian function suppression group; MDR, mammographic breast density reduction; HR, hazard ratio; CI, confidential interval; DFS, disease-free survival; OS, overall survival; RFS, recurrence-free survival; DMFS, distant metastasis-free survival; LRRFS, locoregional-free survival.

**Supplementary Table 7. Prognosis according to mammographic breast density reduction in permanent menopause group.**

| Survival | MDR | 8-year survival rate | Log-rank *P* value |
| --- | --- | --- | --- |
| DFS | MDR-negative (n = 49) | 88.7% | .169 |
|  | MDR-positive (n = 14) | 100% |  |
| OS | MDR-negative (n = 49) | 97.8% | .455 |
|  | MDR-positive (n = 14) | 100% |  |
| RFS | MDR-negative (n = 49) | 90.9% | .209 |
|  | MDR-positive (n = 14) | 100% |  |
| DMFS | MDR-negative (n = 49) | 93.2% | .254 |
|  | MDR-positive (n = 14) | 100% |  |
| LRRFS | MDR-negative (n = 49) | 92.9% | .365 |
|  | MDR-positive (n = 14) | 100% |  |

Abbreviations: MDR, mammographic breast density reduction; DFS, disease-free survival; OS, overall survival; RFS, recurrence-free survival; BCFI, breast cancer-free interval; DMFS, distant metastasis-free survival; LRRFS, locoregional-free survival.

**Supplementary Figure 1.** Breast density reduction (MDR) rate according to the treatment groups in (A) Breast Density Cohort and (B) patients with ER-positive, HER2-negative breast cancer. TAM only, tamoxifen-only group; TAM + OFS, tamoxifen plus ovarian function suppression group.


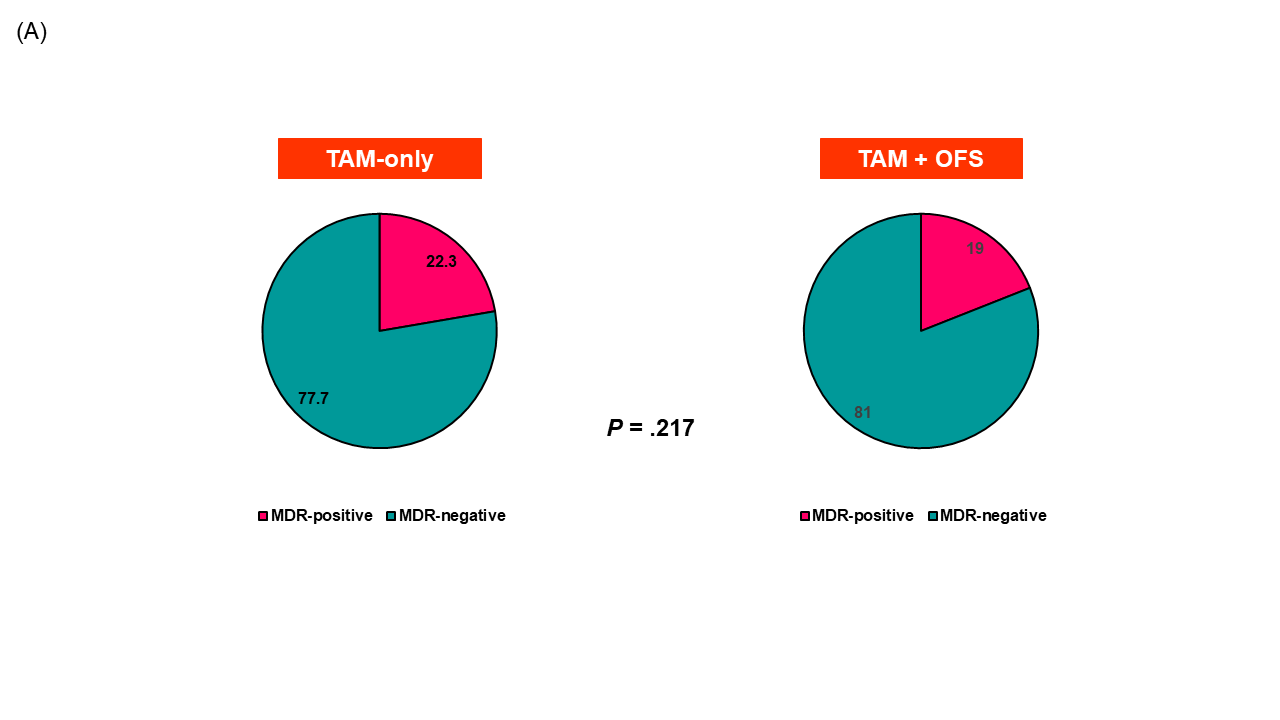


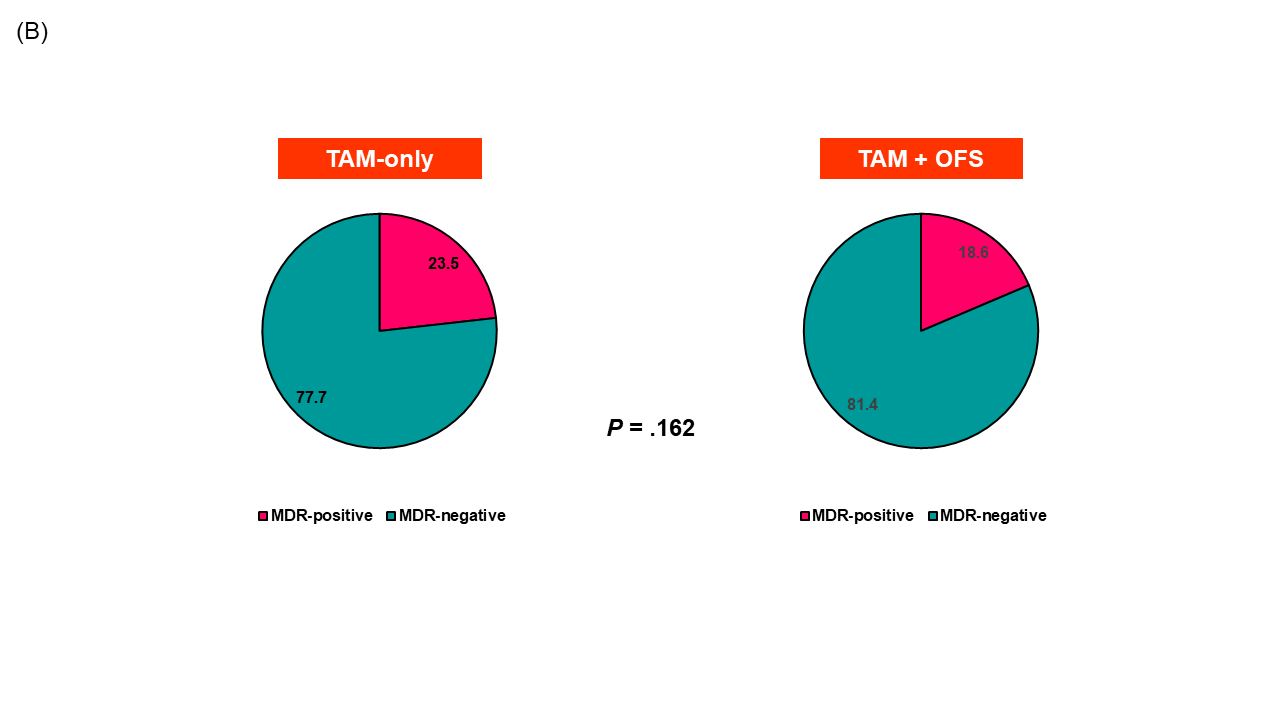

Supplement: Supplementary file 2 [file js9-110-0934-s002.docx]
